# Cash Transfers in the Perinatal Period and Investigations of Infant Maltreatment

**DOI:** 10.1001/jamapediatrics.2026.1602

**Published:** 2026-05-07

**Authors:** Sumit Agarwal, H. Luke Shaefer, Samiul Jubaed, William Schneider, Eric D. Finegood, Mona Hanna

**Affiliations:** 1 Institute for Healthcare Policy and Innovation, University of Michigan Medical School, Ann Arbor; 2 Poverty Solutions, University of Michigan, Ann Arbor; 3 Gerald R. Ford School of Public Policy, University of Michigan, Ann Arbor; 4 School of Social Work, University of Illinois at Urbana-Champaign, Urbana; 5 Michigan State University–Hurley Children’s Hospital Pediatric Public Health Initiative, Charles Stewart Mott Department of Public Health, Michigan State University College of Human Medicine, Flint

## Abstract

**Importance:**

Infants are most vulnerable to child maltreatment, in part due to poverty during the perinatal period. In 2024, the prenatal and infant cash prescription program Rx Kids was launched in Flint, Michigan, achieving nearly 100% aggregate uptake and providing every expectant mother with unconditional cash transfers during pregnancy and infancy.

**Objective:**

To examine whether implementation of the Rx Kids prenatal and infant cash prescription program was associated with changes in child welfare system involvement.

**Design, Setting, and Participants:**

This cross-sectional study used administrative data from Children’s Protective Services in Michigan among infants born January 1, 2021, through December 31, 2024. Synthetic difference-in-differences—a robust method for aggregate-level data and a single treated city—was used to compare changes in outcomes in Flint before and after implementation of Rx Kids relative to the corresponding change in control cities without the program. Data analysis was conducted from March 19, 2025, to March 4, 2026.

**Exposure:**

Flint residence among infants born in 2024 after Rx Kids implementation.

**Main Outcomes and Measures:**

The primary outcome was investigated maltreatment allegations within the first 6 months of life. Secondary outcomes included type of allegation (neglect vs nonneglect) and substantiated allegations.

**Results:**

In the 3 years prior to the implementation of Rx Kids, the proportion of infants with an investigated allegation within the first 6 months of life was 21.7% (646 of 2971 infants) in Flint and 19.5% (3921 of 20 124 infants) among control cities. After implementation of Rx Kids in 2024, the investigated allegation rate decreased to 15.5% (165 of 1065 infants) in Flint, falling below the investigated allegation rate of 20.6% (1303 of 6317 infants) among the control cities. Rx Kids was associated with a statistically significant 7.0–percentage-point decrease (95% CI, −12.9 to −1.0; *P* = .02) in the investigated allegation rate, corresponding to a 32% decrease relative to the preintervention period. There was a decrease in the rate of neglect-related, nonneglect-related, and substantiated allegations; these were directionally consistent with the primary outcome but not statistically significant. Results were robust to alternative model specifications.

**Conclusions and Relevance:**

In this study, the Rx Kids prenatal and infant cash prescription program was associated with a significant reduction in investigated allegations of maltreatment among infants. These findings provide important evidence about the role of economic stability in preventing child welfare system involvement.

## Key Points

**Question:**

Was implementation of the Rx Kids prenatal and infant cash prescription program in Flint, Michigan, associated with changes in Children’s Protective Services (CPS) investigations?

**Findings:**

In this cross-sectional study, the implementation of Rx Kids was associated with a statistically significant 7.0–percentage-point decrease in the investigated allegation rate among infants born in Flint, corresponding to a 32% decrease relative to the preintervention period.

**Meaning:**

Rx Kids was associated with substantial, population-level reductions in investigations of maltreatment among infants in the earliest months of life.

## Teaser

This cross-sectional study examines whether implementation of the Rx Kids prenatal and infant cash prescription program was associated with changes in child welfare system involvement in Flint, Michigan.

## Introduction

Child maltreatment, which includes abuse and neglect, is a pernicious public health problem. More than one-third of children in the US experience an investigation for child maltreatment by their 18th birthday.^1^ Children affected by maltreatment have worse mental and physical health and are more likely to be involved in the criminal justice system, attain lower levels of education, and earn less income into adulthood.^2,3,4,5,6,7^ Each case of nonfatal child maltreatment is estimated to cost $1.1 million in 2025 US dollars, reaching a national economic burden of approximately $585 billion per year,^8^ encompassing costs from health care, the child welfare and criminal justice systems, and other societal impacts.^7^

Poverty is a major risk factor for child maltreatment and involvement with the child welfare system.^9^ Children living in low-income households experience maltreatment at 5 times the rate of other children and are 7 times more likely to experience neglect-specific maltreatment.^10^ Living in communities of concentrated disadvantage also increases maltreatment risk.^11,12^ There are several possible interrelated mechanisms. Poverty increases the material hardships faced by families, including food and housing insecurity, and can limit parents’ ability to purchase essential items for their children. Financial strain also contributes to family stress, which can undermine the capacity of parents to provide a safe, stable, and nurturing home environment, and increases parental risk of mental health challenges, substance use, partner conflict, and domestic violence.^13,14,15,16^ Consequently, a growing body of research finds that income supports in the US, such as refundable tax credits, can prevent child maltreatment.^17,18,19,20,21,22,23,24,25,26,27,28,29,30,31,32,33,34,35^

Infants are the most vulnerable to maltreatment, with a substantiated allegation rate 2 to 4 times greater than other pediatric age groups.^36^ The heightened physical and mental demands of the perinatal period—coupled with the acute economic shock from decreasing income and increasing expenses before and after birth—present unique vulnerabilities that can exacerbate risk factors associated with child maltreatment.^37,38,39,40^ Health-related factors in the perinatal period, such as inadequate prenatal care, low birth weight, and prematurity, also increase the risk for infant maltreatment.^41,42,43^ Whether a time-bound perinatal cash transfer program can prevent investigations into alleged maltreatment remains an open question and one of critical importance given the lifelong trajectories of early life adversity.

In January 2024, Rx Kids launched in Flint, Michigan, a midsized city with one of the nation’s highest child poverty rates. All expectant mothers residing in the city of Flint are eligible for a 1-time transfer of $1500 midpregnancy and $500 monthly after birth until age 1 year. Rx Kids is the first cash transfer program in the US that targets the perinatal period with universal eligibility (ie, communitywide and no means testing based on income or asset thresholds). The program has achieved a high aggregate uptake of nearly 100%,^44,45^ and prior work has demonstrated improved family financial security, use of prenatal care, birth outcomes, and maternal mental health, leading to the hypothesis that the program may be associated with reduced Children’s Protective Services (CPS) involvement.^46,47,48^ Our research tests this hypothesis by using a quasi-experimental study design, the synthetic difference-in-differences method, to examine whether the implementation of Rx Kids was associated with a reduction in investigated allegations.

## Methods

### Data Sources

This cross-sectional study used administrative data from CPS in Michigan for all investigated maltreatment allegations among infants from January 1, 2019, through June 30, 2025. The data were provided to the University of Michigan’s Child and Adolescent Data Lab under an agreement with the Michigan Department of Health and Human Services. The data included demographic information, report date, details on the type of alleged maltreatment, and whether an allegation was substantiated. In our primary analysis, we focused on investigated allegations among infants born January 1, 2021, through December 31, 2024, due to changes in reporting in 2019 and 2020 related to the COVID-19 pandemic.^49,50^ Infants born January 1, 2019, through December 31, 2020, were included in sensitivity analyses described later. The Michigan Department of Health and Human Services also provided statewide data on all births from January 1, 2021, through December 31, 2024, based on birth certificate records, and we imputed or obtained data for birth counts from January 1, 2019, through December 31, 2020, from publicly available records. Data were geocoded based on address to the city level, after which we used a deidentified version of the data for the analysis. We obtained additional data from the 2019-2023 US Census Bureau’s American Community Survey comprising the demographic and socioeconomic characteristics of cities. This study was approved as a retrospective analysis with a waiver of informed consent by the institutional review boards at the University of Michigan and Michigan State University. The Strengthening the Reporting of Observational Studies in Epidemiology (STROBE) reporting guidelines were followed for this cross-sectional study. Data analysis was conducted from March 19, 2025, to March 4, 2026.

### Exposure

To address how poverty influences maternal and infant health, the Rx Kids cash prescription program was launched in Flint, Michigan. Rx Kids began enrolling participants in January 2024. Eligible participants enroll online and must provide documentation of their residency within the city of Flint and pregnancy or childbirth status by medical documentation. After verification, the program provides participants with $1500 during pregnancy (ie, after 20 weeks’ gestation in 2024) and $500 per month for 12 months after birth, totaling $7500 in unconditional cash. The program is administered by the nonprofit GiveDirectly and funded as a private-public partnership. As a nontaxable gift to beneficiaries, the cash transfers are not subject to income taxes and do not affect eligibility for most other public benefits.^51^ No other major changes were identified in local economic conditions, CPS reporting and investigation practices, or perinatal supports and services that would coincide with the timing of Rx Kids implementation in Flint. Given Rx Kids’ nearly 100% uptake,^44,45^ the exposure was defined as Flint residence among infants born in 2024 after Rx Kids implementation.

### Study Measures

The primary study outcome was investigated maltreatment allegations within the first 6 months of life. Investigated allegations were aggregated to the annual level based on the infant’s date of birth, and the investigated allegation rate was calculated using yearly denominators of city birth counts. The outcomes were defined through 6 months of follow-up to ensure complete follow-up for all infants born in 2024, and because most investigations among infants occur during the first 6 months of life.^52^ Secondary outcomes included whether an allegation was substantiated after investigation and whether an allegation was classified as one related to neglect or nonneglect, such as physical, emotional, or sexual abuse.

### Statistical Analysis

We used the quasi-experimental synthetic difference-in-differences method to compare changes in outcomes in Flint before and after Rx Kids implementation relative to the corresponding change in comparable cities without the program.^53,54^ The method is the most appropriate and valid for situations such as ours with a single treated unit and group-level data among a panel of cities. The original synthetic control method was also considered for this analysis,^55,56^ but this method is known to have problems when applied to short panels of data, which can lead to faulty statistical inference.^57,58,59^

Using synthetic difference-in-differences, the synthetic control for Flint was constructed from a donor pool of 21 control cities without Rx Kids that resembled Flint most closely in terms of population size, poverty rate, and racial composition.^47,48^ Using data from the years prior to Rx Kids implementation, the algorithm of synthetic difference-in-differences assigned weights to donor pool cities as well as time-specific weights to generate a synthetic control that follows the same trends in outcomes as Flint in the preintervention period and counterfactually approximates how Flint would have evolved in the postintervention period in the absence of Rx Kids (eAppendix in Supplement 1). An event study approach was used to assess for parallel trends in the preintervention period.^60^ City characteristics, including poverty rate, median household income, racial and educational composition, additional household characteristics (female-headed, renter-occupied, and housing-burdened proportions), and total population, were included as covariates. The coefficient of interest from synthetic difference-in-differences was estimated as the change in outcomes in Flint before and after Rx Kids implementation compared with the corresponding weighted change in control cities without the program.

To examine whether observed changes in Flint could have arisen due to chance, synthetic difference-in-differences uses permutation-based placebo tests, a form of exact inference in which treatment is iteratively reassigned to control cities in the donor pool to generate a reference distribution against which a *P* value can be calculated, particularly in the context of a single treated cluster. All statistical tests were 2-tailed with a level of significance set at *P* < .05. We conducted several sensitivity analyses to probe the robustness of our results to the exclusion of city characteristics as covariates, time aggregation at the half-year level, an alternative donor pool of control cities that was also available in the data consisting of 63 of Michigan’s most populous cities, and the length of the preintervention period. We also examined the results using the original synthetic control method, demonstrating how it leads to faulty inference in settings with a short panel. Finally, we assessed for changes in birth counts or any compositional change in who gives birth and conducted a sensitivity analysis focusing on the number of investigated maltreatment allegations as an outcome. Analyses were conducted using Stata version 19.5 (StataCorp LLC).

## Results

**Figure 1.**
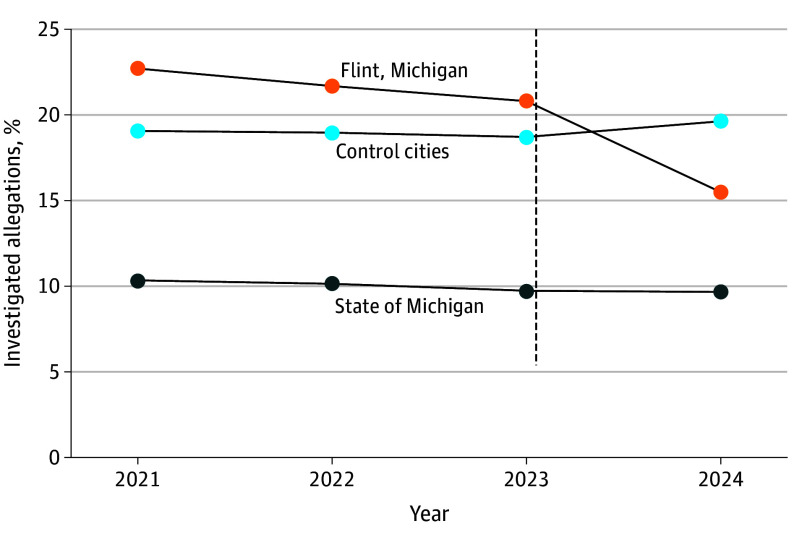
Line Graph of Investigated Allegations Within the First 6 Months of Life for Infants in Flint, Michigan, Control Cities, and the State of Michigan The vertical dashed line differentiates the preintervention period (2021-2023) from the postintervention period (2024).

From January 1, 2021, through June 30, 2025, there were 40 332 investigated allegations of child maltreatment within the first 6 months of life in Michigan among 404 292 infants born January 1, 2021, through December 31, 2024, for a statewide rate of 10.0% during the study period. In the 3 years prior to the implementation of Rx Kids, the proportion of infants with an investigated maltreatment allegation within the first 6 months of life was 21.7% (646 of 2971 infants) in Flint and 19.5% (3921 of 20 124 infants) among control cities; the trend was relatively stable from January 1, 2021, through December 31, 2023 (Table 1). After implementation of Rx Kids in January 2024, the investigated allegation rate decreased to 15.5% (165 of 1065 infants) in Flint, falling below the investigated allegation rate of 20.6% (1303 of 6317 infants) among control cities (Figure 1; eFigure 1 in Supplement 1). The investigated allegation rate in the control cities increased modestly over the same period.

**Table 1.** Baseline Characteristics Before Weighting

Characteristic	%	
	Flint, Michigan^a^	Control cities^a^
Investigated allegations	21.7	18.9 (10.5)
Year		
2021	22.7	19.1 (10.5)
2022	21.7	19.0 (10.6)
2023	20.8	18.7 (10.9)
Substantiated allegations	4.5	3.0 (1.9)
Poverty rate	34.4	26.7 (9.0)
Deep poverty rate	18.1	12.4 (5.9)
Household income, mean, $	36 194	44 552 (10 417)^b^
Racial and ethnic composition^c^		
American Indian or Alaska Native	0.3	0.3 (0.4)
Asian	0.6	1.1 (1.6)
Hispanic	4.5	9.0 (6.0)
Native Hawaiian or Other Pacific Islander	0.0	0.0 (0.1)
Non-Hispanic Black	56.0	45.4 (20.7)
Non-Hispanic White	32.6	38.7 (18.2)
Other	0.5	0.4 (0.6)
Multiple races	5.5	5.1 (1.7)
Educational composition		
<High school diploma	16.7	14.1 (5.4)
≥High school diploma	83.3	85.9 (5.4)
Households		
With children and female head of household	59.3	53.4 (13.2)
Renter-occupied	45.0	43.8 (11.7)
Housing-burdened	54.4	50.5 (6.4)
Gini index, mean	0.48	0.46 (0.06)
Public health insurance	70.5	56.1 (10.5)
Birth counts in 2021-2023, mean, No.	2971	958 (998)
Population, mean, No.	80 835	25 933 (25 802)

^a^ For the column of Flint, Michigan, as a single unit, point values are reported and measures of variance are not applicable. For the 21 control cities, data are presented as mean (SD).

^b^ Data are expressed as the mean (SD) of the 21 control cities’ median household income.

^c^ Race and ethnicity were determined by self-report. Other includes individuals whose race and ethnicity were not captured in the predefined categories in the American Community Survey.

**Figure 2.**
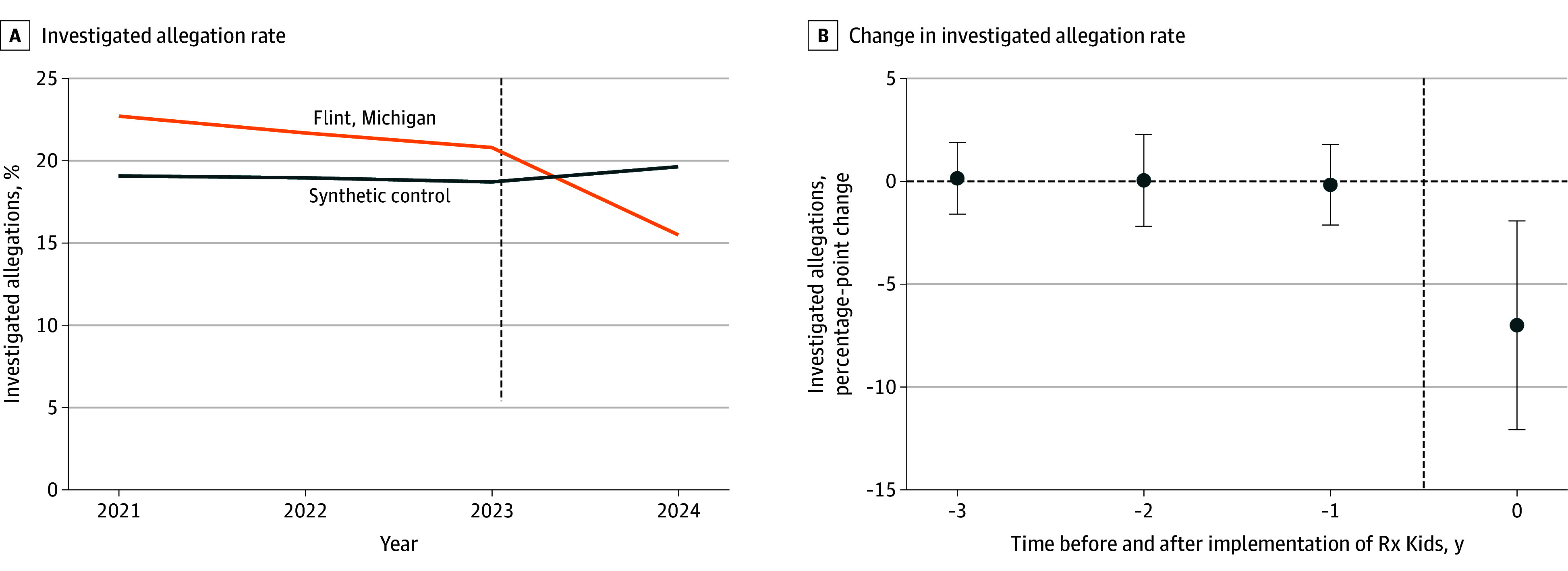
Line Graph of the Investigated Allegation Rate and Event Study of Its Change by Synthetic Difference-in-Differences A, Investigated allegation rate in Flint, Michigan, vs synthetic control. B, Change in the investigated allegation rate, showing point estimates from synthetic difference-in-differences and 95% CIs (error bars) via permutation-based inference. The vertical dashed lines differentiate the preintervention period (2021-2023) from the postintervention period (2024). Synthetic difference-in-differences uses unit- and time-specific weights (see text for details) to achieve balance in preintervention outcomes, focusing on trends rather than absolute levels to establish parallel trends.

Using synthetic difference-in-differences to estimate the change in Flint before and after Rx Kids implementation compared with the corresponding change in control cities without the program, Rx Kids was associated with a statistically significant 7.0–percentage-point decrease (95% CI, −12.9 to −1.0; *P* = .02) in the investigated allegation rate (Figure 2 and Table 2), corresponding to a 32% decrease (95% CI, −59% to −5%) relative to the rate in Flint during the preintervention period. The weights used to generate the synthetic control are presented in eTables 1 and 2 and eFigure 2 in Supplement 1, and trends were parallel in the preintervention period (Figure 2; eFigure 3 in Supplement 1). There was also a decrease in the investigated allegation rate at the half-year level (eFigure 3 in Supplement 1) as well as decreases in the rates of neglect-related and nonneglect-related allegations, similar in magnitude on an absolute basis and a relative basis, and the rate of substantiated allegations; these were directionally consistent with the primary outcome but not statistically significant.

**Table 2.** Estimates From Synthetic Difference-in-Differences for the Primary and Secondary Outcomes

Outcome	Rate in Flint, Michigan, prior to 2024, mean (SD), %	Synthetic difference-in-differences	
		Estimate (95% CI), percentage-point change	*P* value
Investigated allegations	21.7 (1.0)	−7.0 (−12.9 to −1.0)	.02
Neglect-related	10.0 (0.4)	−3.6 (−8.2 to 1.0)	.13
Nonneglect-related	11.7 (0.7)	−3.4 (−9.0 to 2.3)	.24
Substantiated allegations	4.5 (0.5)	−1.3 (−4.2 to 1.6)	.36

In sensitivity analyses, the results were robust to excluding city characteristics as covariates, using an alternative donor pool, and adding 2 additional years to the preintervention period (Table 3). Synthetic difference-in-differences was a more robust and appropriate method compared with the original synthetic control method (eTable 3 in Supplement 1). As a final sensitivity check, we found a 10% increase in the number of births in Flint but no significant compositional change; furthermore, despite an increase in births in Flint concentrated at the beginning of 2024, Rx Kids was associated with a significant reduction in the number of investigated allegations in both halves of 2024 as well as overall for the year (−56.9 [95% CI, −76.4 to −37.3] investigated allegations) (eTable 4 in Supplement 1).

**Table 3.** Sensitivity Analyses

Specification^a^	Investigated allegations	
	Estimate (95% CI), percentage-point change	*P* value
Primary study period, 2021-2024		
Prespecified donor pool	−7.0 (−12.9 to −1.0)	.02
Alternative donor pool	−5.8 (−10.0 to −1.5)	.008
Adding 2 y to preintervention period, 2019-2024		
Prespecified donor pool	−6.3 (−11.7 to −0.9)	.02
Alternative donor pool	−7.9 (−12.5 to −3.4)	.001

^a^ The prespecified donor pool comprised 21 control cities that resembled Flint, Michigan, most closely in terms of population size, poverty rate, and racial composition. The alternative donor pool comprised 63 of the most populous cities in Michigan.

## Discussion

Rx Kids, the nation’s first communitywide, unconditional prenatal and infant cash transfer program, was associated with a substantial population-level decline in the proportion of infants with an investigated maltreatment allegation within the first 6 months of life. Prior to Rx Kids implementation, the investigated allegation rate in Flint was more than double the state rate (21.7% vs 10.0%, respectively). After Flint’s rate decreased to 15.5% in 2024, this disparity between the city and the state narrowed by 50%. Rx Kids prevented an estimated 57 infants from being involved in the child welfare system. These findings represent a consequential prevention of early-life adversity with broad implications for children’s health and well-being.

These results are consistent with those of prior studies showing that economic support can prevent child maltreatment.^17,18,19,20,21,22,23,24,25,26,27,28,29,30,31,32,33,34,35^ Rittenhouse^23^ found that an additional $1000 in tax benefits during infancy led to a 3% decline in the number of CPS referrals, investigations, and substantiations by age 3 years among low-income households. Bullinger et al^24^ leveraged year-to-year variation in the Alaska Permanent Fund Dividend, finding that an additional $1000 in dividend payments led to a 10% reduction in unsubstantiated cases of child maltreatment and a 15% reduction in substantiated cases by age 3 years. The magnitude of our estimates, scaled for the size of the Rx Kids cash transfer ($4500 by age 6 months) and assuming the outcomes persist beyond 6 months of age, is closer to that found by Bullinger et al,^24^ possibly due to Rx Kids and the Alaska Permanent Fund Dividend both being universal in design with high uptake. Our findings, consistent with prior literature, support policy efforts focused on providing concrete economic support to prevent child welfare system involvement, most notably embodied by the Family First Prevention Services Act of 2018.^61^

Rx Kids may have prevented maltreatment investigations through multiple pathways. Poverty is a risk factor for CPS referral and increasingly recognized as causally linked to child maltreatment.^15^ It may also be comorbid with a range of factors that lead to maltreatment, including behavioral health conditions.^62^ A prior study of Rx Kids showed that the program was associated with reductions in food insecurity, housing instability, and other financial hardships.^46^ In fact, Rx Kids led to a near elimination of postpartum evictions among eligible mothers, consistent with evidence from eviction moratoria during the COVID-19 pandemic that reduced county-level rates of child maltreatment.^63^ The Rx Kids program was also associated with improvements in maternal mental health and parental stress.^46,64,65^ The reductions in financial strain and parental stress can in turn reduce the risk of child maltreatment.^66,67,68^ Furthermore, premature and low-birth-weight infants have the highest risk of maltreatment,^41^ and Rx Kids reduced the rate of these adverse birth outcomes.^48^

A notable strength of our study is the use of the synthetic difference-in-differences method, which combines attractive features of both the original synthetic control method and the difference-in-differences approach. Synthetic difference-in-differences is an extension of the synthetic control method that similarly reweights control cities,^59^ but it differs from the synthetic control method in its use of both unit-specific and time-specific weights to achieve balance in preintervention outcomes. It focuses on trends rather than absolute levels to establish parallel trends, a key causal assumption of the difference-in-differences framework. Because it reweights in 2 dimensions, synthetic difference-in-differences possesses a form of double robustness, reducing the chance for bias from unobserved factors. In addition, its use of fixed effects absorbs additive differences across units and time, further strengthening its robustness relative to the original synthetic control method. Simulation results show that synthetic difference-in-differences dominates other estimators, including the synthetic control method.^53^ These features of synthetic difference-in-differences improve statistical power, limit researcher degrees of freedom, and make it less sensitive to the donor pool’s composition and better suited to panels with few preintervention periods.^69^

### Limitations

This study has several limitations. First, because Rx Kids was not allocated randomly, there may be residual confounding unaccounted for by our quasi-experimental study design. The use of both unit and time weights establishes parallel trends to ensure the plausibility of the difference-in-differences strategy; it is analogous to techniques such as the adjustment of covariates to address the parallel-trends assumption.^52^ Relatedly, the primary outcome from our administrative data, investigated allegations of child maltreatment, reflects not only the actual occurrence of maltreatment but also reporting practices and CPS capacity, screening, and investigation practices; this raises the possibility of confounding if there are concurrent and differential changes in CPS capacity or practices. The child welfare system in Michigan, however, is centrally administered at the state level, so all counties adhere to the same set of standards and practices. Based on discussions with local and statewide experts, we are not aware of differential changes related to CPS in Flint relative to control cities that coincided with the implementation of Rx Kids. There were also no other major differential changes related to emergence from the COVID-19 pandemic. The synthetic difference-in-differences strategy nonetheless relies to some extent on the common-shocks and no-concurring-interventions assumptions. We are similarly not aware of concurrent interventions specifically in the control cities, but if present, this would bias our results toward the null. Because our data only included infants, we were unable to implement a falsification test, such as investigated allegations among older children, that would allow us to probe these assumptions further. Older children also represent an area for future research given the potential for spillover benefits among siblings.

Second, our primary analysis focused on births between 2021 and 2024, a relatively short study duration with a single postperiod year. The results, however, were robust to the inclusion of 2 additional years of preperiod data. Furthermore, trends were stable in the preintervention period, with each data point for Flint representing approximately 1000 births, which limits the influence of random fluctuation. We are unable to demonstrate sustainability of the results with a single year of postperiod data. Part of our preintervention period coincided with the COVID-19 pandemic; there could be residual pandemic effects that may limit generalizability, although the investigated allegation rate in Flint in 2019 was only modestly higher compared with the preintervention part of our primary study period. Third, we defined exposure as Flint residence among infants born in 2024 after Rx Kids implementation rather than actual enrollment in the program. Our analysis thus followed the intention-to-treat principle to capture the population-level outcome of Rx Kids, a parameter of key interest to policymakers. Due to the high uptake rates of Rx Kids, our intention-to-treat estimates are likely very close to estimates for the program itself. Defining exposure based on residence also assumes that the city in which an infant resides at the time of an investigated allegation 6 months into life is the same city in which the mother resided when the infant was born. This could produce outcome measurement error if there is migration into or out of communities. Fourth, there was evidence of a slight increase in births without compositional change in Flint in 2024 compared with prior years, which could mechanically reduce the investigated allegation rate. Despite the increase in births, we found a significant decline in the absolute count of investigated allegations. Fifth, our analysis may not have been well powered for the secondary outcomes in which we observed nonsignificant declines in the subcategories of investigated allegations. These were nonetheless directionally consistent with the primary outcome, and we follow the literature in focusing on investigated allegations as a principal outcome as opposed to substantiated allegations given that the latter is a rarer outcome, may be sensitive to CPS capacity or practices, and has not been shown to be a better predictor of risk compared with unsubstantiated cases.^70^ Investigated allegations may be sensitive to ascertainment bias, although Rx Kids was not specifically implemented or publicized as an effort to prevent child maltreatment. Sixth, the long-term implications of reducing investigations of child maltreatment during the first 6 months of life remain unknown, but preventing an initial investigation likely reduces the risk of future investigations.^71^

## Conclusions

This cross-sectional study found that Rx Kids, designed as a universal and unconditional prenatal and infant cash prescription program to reduce perinatal poverty and improve health outcomes, yielded cross-sector benefits extending to child welfare system involvement. Rx Kids was associated with substantial, population-level reductions in investigations of maltreatment among infants in the earliest months of life. These findings underscore the importance of providing economic support during the perinatal period to improve child health and well-being.

## Notes

Supplement 1. **eAppendix.** Supplementary Methods **eTable 1.** Unit Weights for the Primary Outcome (Investigated Allegations) in the Synthetic Difference-in-Differences Analysis **eTable 2.** Time Weights for the Primary Outcome (Investigated Allegations) in the Synthetic Difference-in-Differences Analysis **eTable 3.** Results From the Original Synthetic Control Method With a Short Panel **eTable 4.** Results From Synthetic Difference-in-Differences for Composition of Births, Number of Births, and Number of Investigated Allegations **eFigure 1.** Investigated Allegation Rate in Flint and All Control Cities **eFigure 2.** Preintervention Balance Across Control Units From Synthetic Difference-in-Differences for the Primary Outcome (Investigated Allegations) **eFigure 3.** Change in the Investigated Allegation Rate by Half-Year
eReferences

Supplement 2. Data Sharing Statement
