# Supplementary material for: Cash Transfers in the Perinatal Period and Investigations of Infant Maltreatment
Source: JAMA Pediatr. 2026 May 7;180(8):876–83. doi: 10.1001/jamapediatrics.2026.1602 (PMC13154033; doi:10.1001/jamapediatrics.2026.1602)
Supplement: Supplement 1. — eAppendix. Supplementary Methods eTable 1. Unit Weights for the Primary Outcome (Investigated Allegations) in the Synthetic Difference-in-Differences Analysis eTable 2. Time Weights for the Primary Outcome (Investigated Allegations) in the Synthetic Difference-in-Differences Analysis eTable 3. Results From the Original Synthetic Control Method With a Short Panel eTable 4. Results From Synthetic Difference-in-Differences for Composition of Births, Number of Births, and Number of Investigated Allegations eFigure 1. Investigated Allegation Rate in Flint and All Control Cities eFigure 2. Preintervention Balance Across Control Units From Synthetic Difference-in-Differences for the Primary Outcome (Investigated Allegations) eFigure 3. Change in the Investigated Allegation Rate by Half-Year eReferences [file jamapediatr-e261602-s001.pdf]

Supplemental Online Content

Agarwal S, Shaefer HL, Jubaed S, Schneider W, Finegood ED, Hanna M. Cash transfers in the perinatal period and investigations of infant maltreatment. *JAMA Pediatr*. Published online May 7, 2026. doi:10.1001/jamapediatrics.2026.1602

eAppendix. Supplementary Methods
eTable 1. Unit Weights for the Primary Outcome (Investigated Allegations) in the Synthetic Difference-in-Differences Analysis
eTable 2. Time Weights for the Primary Outcome (Investigated Allegations) in the Synthetic Difference-in-Differences Analysis
eTable 3. Results From the Original Synthetic Control Method With a Short Panel
eTable 4. Results From Synthetic Difference-in-Differences for Composition of Births, Number of Births, and Number of Investigated Allegations
eFigure 1. Investigated Allegation Rate in Flint and All Control Cities
eFigure 2. Preintervention Balance Across Control Units From Synthetic Difference-in-Differences for the Primary Outcome (Investigated Allegations)
eFigure 3. Change in the Investigated Allegation Rate by Half-Year
eReferences

This supplemental material has been provided by the authors to give readers additional information about their work.

© 2026 Agarwal S et al. *JAMA Pediatrics*.

eAppendix: Supplementary Methods

Because the city of Flint has one of the highest child poverty rates (59% in 2023) and largest proportions of Black residents (56%) in the state—factors that are highly correlated with CPS reporting and involvement—we restricted the pool of potential control cities based on prespecified criteria, which led to a donor pool of 21 control cities. The criteria for inclusion in the donor pool of control cities from elsewhere in Michigan included cities with a population of 5,000 to 125,000, a poverty rate of greater than or equal to 15%, and a non-Hispanic Black population of at least 20%, as determined using the U.S. Census Bureau's 2019-2023 American Community Survey. After excluding areas adjacent to the city of Flint such as Beecher and Flint Township as well as Kalamazoo, which had its own perinatal programming during our study period, the twenty-one cities that met the criteria included Albion, Benton Harbor, Benton Township, Bridgeport Township, Buena Vista Township, Eastpointe, Ecorse, Harper Woods, Highland Park, Inkster, Jackson, Lansing, Muskegon, Muskegon Heights, Pontiac, River Rouge, Saginaw, St. Louis (in Michigan), Wayne, Ypsilanti, and Ypsilanti Township.

A potential concern with this donor pool, despite being prespecified, is that it includes a relatively small number of cities as well as a few relatively small-sized cities. We conducted a sensitivity analysis using a distinct donor pool of control cities that was also available in the data, specifically 63 of the most populous cities in Michigan excluding townships and similarly excluding Kalamazoo as above. These 63 cities included: Adrian, Allen Park, Ann Arbor, Auburn Hills, Battle Creek, Bay City, Birmingham, Burton, Canton, Clinton, Dearborn, Dearborn Heights, Detroit, East Lansing, Eastpointe, Farmington Hills, Ferndale, Forest Hills, Garden City, Grand Rapids, Hamtramck, Holland, Holt, Inkster, Jackson, Kentwood, Lansing, Lincoln Park, Livonia, Madison Heights, Marquette, Midland, Monroe, Mount Pleasant,

© 2026 Agarwal S et al. *JAMA Pediatrics*.

Muskegon, Norton Shores, Novi, Oak Park, Okemos, Pontiac, Port Huron, Portage, Redford, Rochester Hills, Romulus, Roseville, Royal Oak, Saginaw, Saint Clair Shores, Shelby, Southfield, Southgate, Sterling Heights, Taylor, Troy, Walker, Warren, Waterford, Waverly, Westland, Wyandotte, Wyoming, and Ypsilanti. This was not our preferred donor pool specification because the criterion of the largest cities diverged from the prespecified criteria used in prior Rx Kids studies, and it leads to a donor pool substantially different than Flint on key dimensions that are related to child maltreatment. For example, the median poverty rate of these 63 cities was 11.6% (which is different than Flint by almost a factor of three), and the median proportion of non-Hispanic Black residents was 8.2% (which is different than Flint by nearly a factor of seven). This is particularly important when using the synthetic control method, as discussed in further detail in eTable 3. The features of synthetic difference-in-differences (see main text) make it less sensitive to the composition of the donor pool.

Unit and time weights were estimated through a regularized optimization procedure,[53] which was implemented using the sdid and sdid_event packages in Stata.[54] In short, the optimization procedure determines (1) unit weights that balance the preintervention trend in the outcome, residualized for the covariates, among the unexposed units with that of the exposed unit and (2) time weights that balance pre- and postintervention periods for the outcome among the unexposed units. Regularization ensures well-dispersed, stable weights and prevents overfitting. These weights are then used in a weighted two-way fixed effects regression of difference-in-differences.

© 2026 Agarwal S et al. *JAMA Pediatrics*.

eTable 1: Unit Weights for the Primary Outcome (Investigated Allegations) in the Synthetic Difference-in-Differences Analysis

Donor City	Weights
Albion	0.04651482
Benton Harbor	0.07667844
Benton Township	0.03837185
Bridgeport Township	0.05365189
Buena Vista Township	0.04815389
Eastpointe	0.05666218
Ecorse	0.0606078
Harper Woods	0.04317521
Highland Park	0.02471664
Inkster	0.0159125
Jackson	0.04504911
Lansing	0.04650577
Muskegon	0.03825902
Muskegon Heights	0.03533298
Pontiac	0.03159653
River Rouge	0.07526972
Saginaw	0.05019247
St. Louis	0.06235585
Wayne	0.04630271
Ypsilanti	0.04773139
Ypsilanti Township	0.05695925

© 2026 Agarwal S et al. *JAMA Pediatrics*.

eTable 2: Time Weights for the Primary Outcome (Investigated Allegations) in the Synthetic Difference-in-Differences Analysis

Year	Time Weights
2021	0.43497877
2022	0.14212496
2023	0.42289627

© 2026 Agarwal S et al. *JAMA Pediatrics*.

eTable 3: Results From the Original Synthetic Control Method With a Short Panel

	Investigated Allegations			
**Specification**	**Estimate**	**RMSPE Ratio for Flint**	**Range of RMSPE Ratios from Placebo Tests**	***P* value**
Primary study period (2021-2024)				
Prespecified donor pool (21 cities)	-6.9 pp	800 billion	0.29 to 5.35	0
Alternative donor pool (63 cities)	-7.1 pp	38 billion	0.27 to 4.48 trillion	0.38
Adding two additional years to the preintervention period (2019-2024)				
Prespecified donor pool (21 cities)	-7.4 pp	7.78	0.17 to 3.27	0
Alternative donor pool (63 cities)	-7.7 pp	6.59	0.31 to 519 billion	0.46

Notes: All estimates are interpreted as a percentage-point (pp) change. The results using the original synthetic control method show effect sizes that are similar across all specifications. Flint had the largest or second largest reduction in the investigated allegation rate compared to the placebo tests for all other units in the prespecified and alternative donor pools. Statistical inference under the synthetic control method, however, can be unreliable in the setting of few preintervention periods and many cities in the donor pool. In the synthetic control method, p-values are determined by comparing the root mean square prediction error (RMSPE) ratio for Flint against a reference distribution of RMSPE ratios from placebo tests.[1-3] Each ratio in the distribution is calculated as the root mean squared prediction error in the postintervention period (the post-RMSPE) divided by the root mean squared prediction error in the preintervention period (the pre-RMSPE). For the 1st, 2nd, and 4th specifications in the table above, RMSPE ratios range from less than one to the billions and trillions, driven by the denominator (pre-RMPSE); this is evidence of overfitting to idiosyncratic noise, the risk of which increases with short panels of data and large donor pools.[4,5] The synthetic control method is not appropriate or valid in such a setting; in fact, there is no effect size for Flint large enough to be statistically significant, implying 0% statistical power. Extending the preintervention period, when possible, and trimming the donor pool to units similar to the treated unit in the preintervention period can correct this issue of overfitting, as suggested by the 3rd specification listed in the table that used 5 years in the preintervention period and a smaller group of donor units more similar to Flint (i.e., the 21 control cities in the prespecified donor pool that resembled Flint most closely in terms of population size, poverty rate, and racial composition). Synthetic difference-in-differences has several features that improve upon known limitations of the synthetic control method (see main text) and was thus our primary method for studying the effect of Rx Kids on child welfare system involvement.

© 2026 Agarwal S et al. *JAMA Pediatrics*.

eTable 4: Results From Synthetic Difference-in-Differences for Composition of Births, Number of Births, and Number of Investigated Allegations

	Synthetic Difference-in-Differences	
**Outcome**	**Estimate (95% CI)**	***P* value**
Maternal age: 16-26	-1.6 pp<br>(-12.6 to 9.3)	0.77
Non-Hispanic Black race	3.3 pp<br>(-9.9 to 16.5)	0.62
Maternal education: less than high school	1.0 pp<br>(-9.6 to 11.7)	0.85
Not married	2.4 pp<br>(-5.6 to 10.4)	0.56
Medicaid as payor	3.5 pp<br>(-15.9 to 22.9)	0.72
First birth (i.e., parity of 0)	1.9 pp<br>(-8.2 to 12.0)	0.71
Number of births	96.7<br>(35.0 to 158.4)	0.002
First half of 2024	75.4<br>(49.0 to 101.8)	< 0.001
Second half of 2024	17.2<br>(-8.4 to 42.9)	0.19
Number of investigated allegations	-56.9<br>(-76.4 to -37.3)	< 0.001
First half of 2024	-30.1<br>(-40.6 to -19.6)	< 0.001
Second half of 2024	-24.2<br>(-33.6 to -14.7)	< 0.001

Notes: There was no statistically significant change in the composition of who gives birth using synthetic difference-in-differences. Furthermore, the point estimates for several of these would, if anything, suggest higher risk of investigated allegations in the postperiod from any potential compositional change. As shown in prior work (reference 48 in the main text), there was similarly no statistically significant change in the composition of who gives birth using individual-level data from birth certificates and a difference-in-differences approach. There was a statistically significant increase in the number of births in Flint concentrated in the first half of 2024; despite an increase in births in Flint, Rx Kids was associated with a significant reduction in the number of investigated allegations in both halves of 2024 as well as overall for the year.

© 2026 Agarwal S et al. *JAMA Pediatrics*.

eFigure 1: Investigated Allegation Rate in Flint and All Control Cities

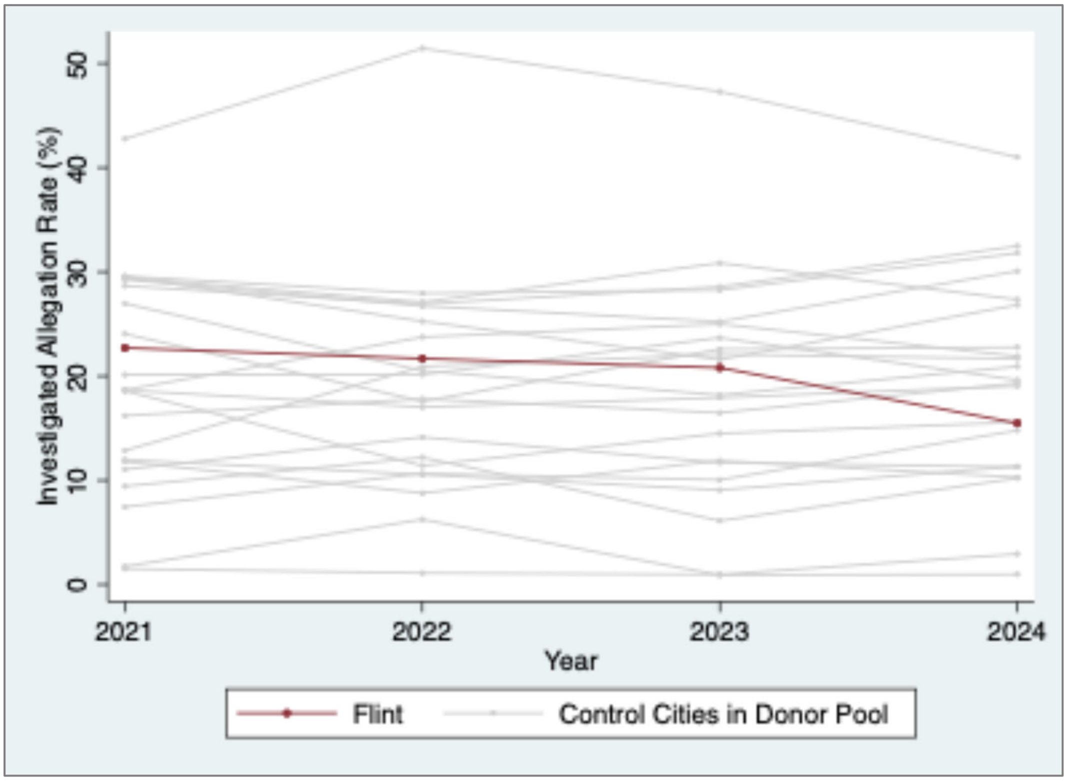

© 2026 Agarwal S et al. *JAMA Pediatrics*.

eFigure 2: Preintervention Balance Across Control Units From Synthetic Difference-in-Differences for the Primary Outcome (Investigated Allegations)

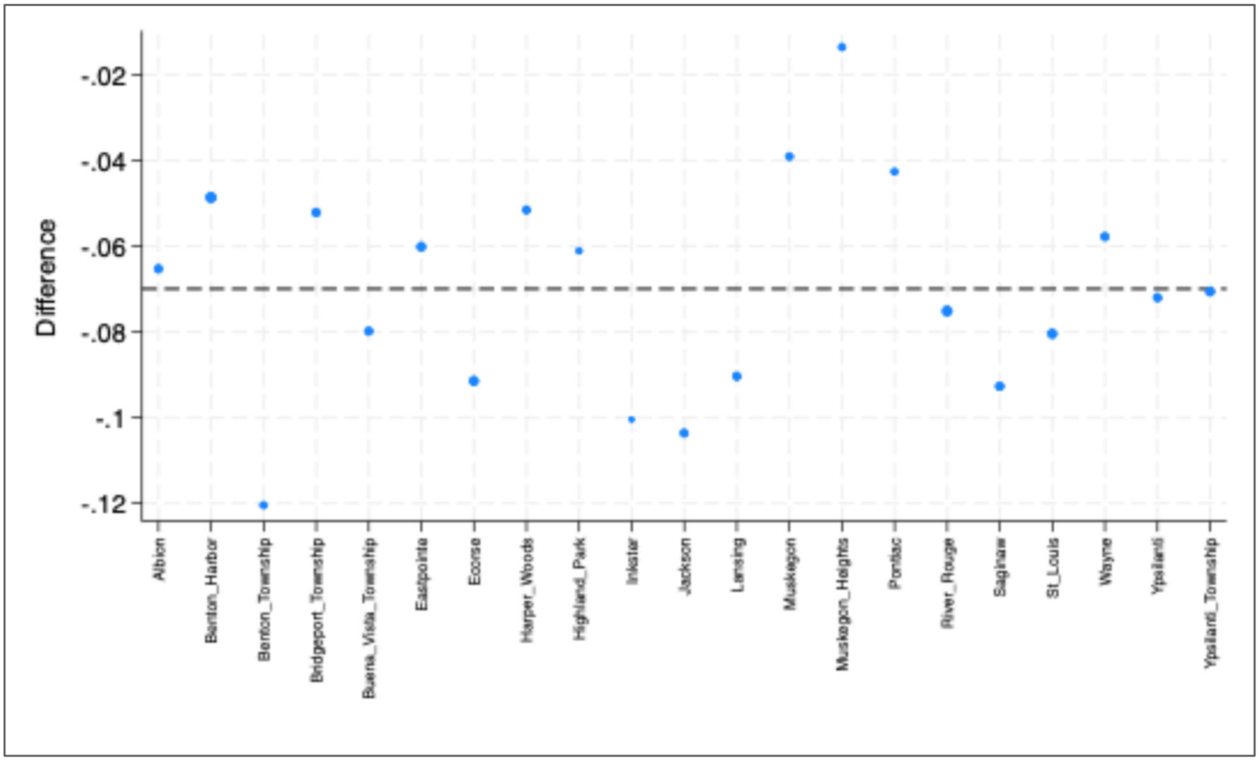

Note: The raw unit and time weights from synthetic difference-in-differences are shown in eTables 1 and 2. This plot shows how well each control unit's preintervention outcomes align with Flint after optimizing the time weights.

© 2026 Agarwal S et al. *JAMA Pediatrics*.

eFigure 3: Change in the Investigated Allegation Rate by Half-Year

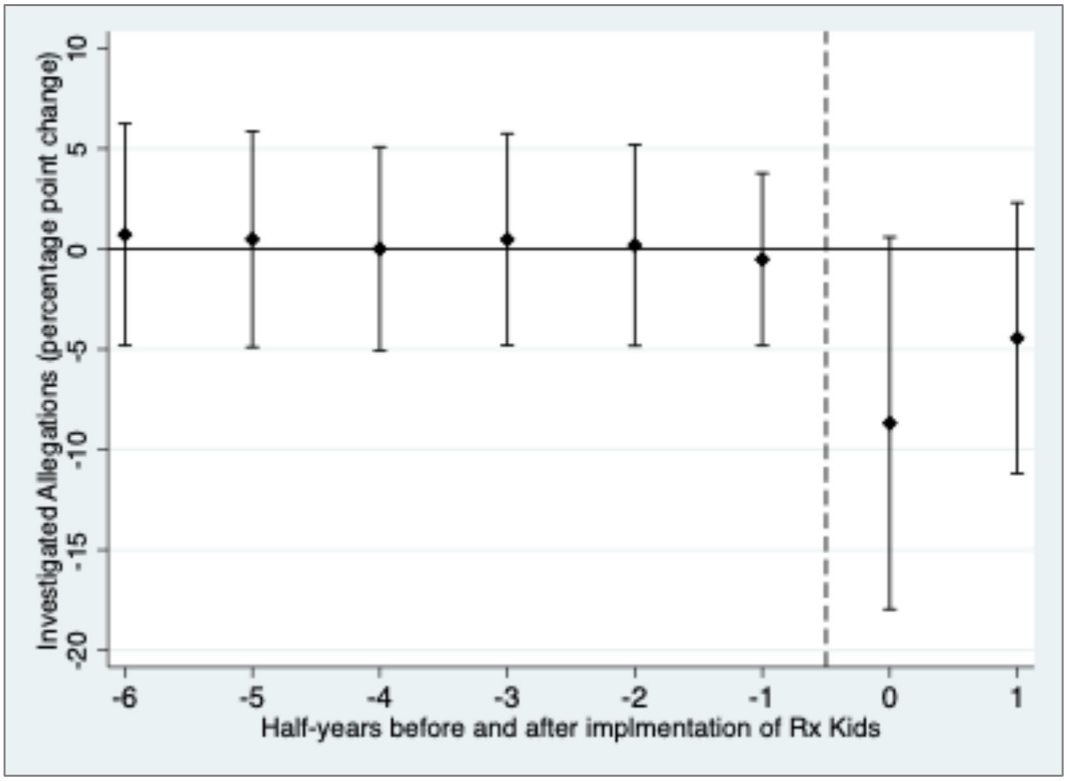

Note: There were decreases in the investigated allegation rate in both the first and second halves of 2024. As subdivided versions of the primary outcome, these estimates had wider confidence intervals that cross zero and are thus statistically insignificant but are directionally consistent with the primary outcome aggregated at the annual level. Estimates for the components of the investigated allegation rate (i.e., birth counts and number of allegations) in the first and second half of 2024 are shown in eTable 4.

© 2026 Agarwal S et al. *JAMA Pediatrics*.

eReferences

1. Abadie A. Using Synthetic Controls: Feasibility, Data Requirements, and Methodological Aspects. *Journal of Economic Literature*. 2021;59(2):391-425. doi:10.1257/jel.20191450
2. Abadie A, Diamond A, Hainmueller J. Synthetic Control Methods for Comparative Case Studies: Estimating the Effect of California's Tobacco Control Program. *Journal of the American Statistical Association*. 2010;105(490):493-505. doi:10.1198/jasa.2009.ap08746
3. Abadie A, Diamond A, Hainmueller J. Comparative Politics and the Synthetic Control Method. *American Journal of Political Science*. 2015;59(2):495-510. doi:10.1111/ajps.12116.
4. Abadie A, Vives-i-Bastida J. Synthetic Controls in Action. arXiv. Preprint posted online March 11, 2022. doi:10.48550/arXiv.2203.06279
5. Hollingsworth A, Wing C. Tactics for Design and Inference in Synthetic Control Studies: An Applied Example Using High-Dimensional Data. Social Science Research Network. Preprint posted online May 3, 2020. doi:10.2139/ssrn.3592088

© 2026 Agarwal S et al. *JAMA Pediatrics*.
