# Supplementary material for: Cash Transfers in the Perinatal Period and Investigations of Infant Maltreatment
Source: JAMA Pediatr. 2026 May 7;180(8):876–83. doi: 10.1001/jamapediatrics.2026.1602 (PMC13154033; doi:10.1001/jamapediatrics.2026.1602)
Supplement: Supplement 2. — Data Sharing Statement [file jamapediatr-e261602-s002.pdf]

Data Sharing Statement

Agarwal. Cash Transfers in the Perinatal Period and Investigations of Infant Maltreatment.
JAMA Pediatr. Published May 07, 2026. doi:10.1001/jamapediatrics.2026.1602

Data
Data available: No
